# Supplementary material for: Folate Pathway Gene Single Nucleotide Polymorphisms and Neural Tube Defects: A Systematic Review and Meta-Analysis
Source: J Pers Med. 2022 Sep 29;12(10):1609. doi: 10.3390/jpm12101609 (PMC9605131; doi:10.3390/jpm12101609)
Supplement: Supplementary file 1 [file jpm-12-01609-s001.zip › Supplementary Table S1.pdf]

Table S1. Study Characteristics.

First Author/ Year	Country	Ethnicity	NTDs Type	SOC	Genotyping Methods	Case/Control	Cases			Controls			
							Heterozygous	homozygous 1	homozygous 2	Heterozygous	homozygous 1	homozygous 2	HW-P value
MTHFR C677T													
van der Put 1998	Netherlands	Caucasian	SB	HB	RFLP-PCR	55/207	22	26	7	111	86	10	0.1927
De Franchis 1998	Italy	Caucasian	SB	HB	RFLP-PCR	203/583	62	89	52	173	313	97	0.0258
Boduroƒülu 1998	Turkey	Caucasian	Mixed	HB	RFLP-PCR	49/93	20	25	4	47	39	7	0.7791
Garcia-Fragoso 2002	USA	Caucasian	Mixed	HB	RFLP-PCR	31/100	10	18	3	41	50	9	0.2539
Perez 2003	Brazil	Mixed	SB	HB	RFLP-PCR	131/126	59	62	10	68	45	13	0.1869
Relton 2003	UK	Caucasian	Mixed	HB	RFLP-PCR	200/578	92	78	30	267	247	64	0.5467
Sadewa 2004	Indonesia	Asian	E	PB	PCR-DHPLC	13/47	12	1	0	39	8	0	0.5236
Kirke 2004	Ireland	Caucasian	Mixed	PB	NS	395/848	151	171	73	439	326	83	0.0523
Gao 2009	China	Asian	Mixed	HB	RFLP-PCR	48/67	4	11	33	16	30	21	0.4156
Eser 2010	Turkey	Caucasian	SB	PB	MCA	39/34	18	14	7	19	17	8	0.2437
Harisha 2010	India	Asian	Mixed	HB	RFLP-PCR	45/102	28	12	5	87	14	1	0.6098
Wei 2013	China	Asian	SB	NS	NS	69/129	22	34	13	39	69	21	0.3013
Liu 2013	China	Asian	Mixed	PB	RFLP-PCR	51/51	10	25	16	22	22	7	0.6916
Qin 2014	China	Asian	Mixed	PB	SNaPshot	189/402	27	90	72	85	195	122	0.6655
Yu 2014	China	Asian	Mixed	PB	NGS	269/189	35	130	104	39	98	52	0.564
Pardo 2014	Chile	Mixed	MM	FB	TaqMan	105/105	59	40	6	59	39	7	0.8718
Wang 2015	China	Asian	Mixed	HB	MassARRAY	144/300	31	40	73	96	45	159	0
Dutta 2017	India	Asian	AE	HB	RFLP-PCR	40/80	26	14	0	61	17	2	0.542
Fang 2018	China	Asian	Mixed	PB	MassARRAY	152/169	21	67	64	40	77	52	0.274
MTHFR A1298C													

van der Put 1998	Netherlands	Caucasian	SB	HB	RFLP-PCR	86/403	37	41	8	179	186	38	0.2981
Stegmann 1999	Germany	Caucasian	SB	PB	ARMS-RFLP	148/174	66	73	9	88	68	18	0.3735
Barber 2000	USA	Caucasian	Mixed	PB	DF	19/85	15	4	0	56	25	4	0.5805
Volcik 2000	USA	Caucasian	MM	PB	TaqMan	233/82	168	61	4	54	25	3	0.9597
Richter 2001	Germany	Caucasian	Mixed	PB	RFLP-PCR	184/233	85	87	12	123	85	25	0.083
Cunha 2002	Brazil	Mixed	Mixed	PB	RFLP-PCR	25/75	14	10	1	42	28	5	0.9093
De Marco 2002	Italy	Caucasian	Mixed	PB	RFLP-PCR	203/202	75	99	29	114	76	12	0.887
Parle-McDermott 2003	Ireland	Caucasian	Mixed	HB	RFLP-PCR	277/256	149	100	28	133	94	29	0.0539
Perez 2003	Brazil	Mixed	SB	HB	RFLP-PCR	131/126	80	43	8	78	41	7	0.6023
Felix 2004	Brazil	Mixed	Mixed	HB	TaqMan	41/44	21	19	1	25	17	2	0.6748
Gos 2004	Poland	Caucasian	Mixed	PB	RFLP-PCR	20/262	7	7	6	118	120	24	0.4057
Relton 2003	UK	Caucasian	Mixed	HB	RFLP-PCR	194/584	91	80	23	279	235	70	0.0628
Sadewa 2004	Indonesia	Asian	FE	PB	PCR-DHPLC	13/47	3	6	4	21	20	6	0.7193
Grandone 2006	Italy	Caucasian	Mixed	HB	RFLP-PCR	15/143	8	7	0	63	69	11	0.1801
Gonzalez-Herrera 2007	Mexico	Mixed	Mixed	PB	RFLP-PCR	108/120	86	21	1	94	25	1	0.6353
Behunova 2010	Slovakia	Caucasian	Mixed	HB	RFLP-PCR	92/290	36	50	6	129	121	40	0.1799
Eser 2010	Turkey	Caucasian	SB	PB	qRT-PCR	39/34	19	3	17	14	12	8	0.1135
Selvi 2010	Turkey	Caucasian	Mixed	FB	qRT-PCR	50/50	25	21	4	23	18	9	0.1219
Pardo 2014	Chile	Mixed	MM	FB	TaqMan	105/105	67	37	1	63	40	2	0.1241
Wang 2015	China	Asian	Mixed	HB	MassARRAY	144/300	76	13	55	126	45	129	0
Yildiz 2016	Turkey	Caucasian	Mixed	FB	qRT-PCR	33/48	15	2	16	18	14	16	0.004
Dutta 2017	India	Asian	AE	HB	RFLP-PCR	40/80	21	9	10	53	21	6	0.0761
MTR A2756G													
van der Put 1997	Netherlands	Caucasian	Mixed	PB	RFLP-PCR	56/364	41	14	1	258	94	12	0.347
Morrison 1998	UK	Caucasian	Mixed	HB	ARMS-RFLP	36/72	22	12	2	48	23	1	0.3365

Morrison 1998	UK	Caucasian	Mixed	HB	RFLP-PCR	32/76	19	12	1	53	20	3	0.53
Christensen 1999	Canada	Caucasian	Mixed	HB	TaqMan	55/97	38	17	0	59	28	10	0.0268
Shaw 1999	USA	Caucasian	Mixed	PB	RFLP-PCR	95/160	64	30	1	104	49	7	0.6897
Johanning 2000	USA	Caucasian	Mixed	HB	qRT-PCR	77/84	59	18	0	70	13	1	0.6576
De Marco 2002	Italy	Caucasian	Mixed	PB	qRT-PCR	174/210	138	34	2	148	61	1	0.0438
Gos 2004	Poland	Caucasian	Mixed	NA	RFLP-PCR	20/262	5	15	0	149	109	4	0.0012
O'Leary 2005	Ireland	Caucasian	Mixed	PB	RFLP-PCR	382/487	235	133	14	310	156	21	0.8067
Doudney 2009	UK	Caucasian	Mixed	PB	qRT-PCR	231/188	147	76	8	118	60	10	0.5167
MTRR A66G													
Wilson 1999	Canada	Caucasian	Mixed	HB	TaqMan	56/97	9	28	19	24	44	29	0.3735
Pietrzyk 2003	Poland	Caucasian	Mixed	PB	RFLP-PCR	104/100	44	49	11	66	29	5	0.4455
Gos 2004	Poland	Caucasian	Mixed	NA	qRT-PCR	20/262	2	16	2	33	158	71	0.0002
Relton 2004	UK	Caucasian	Mixed	PB	RFLP-PCR	201/601	23	125	53	28	265	308	0.002
O'Leary 2005	Ireland	Caucasian	Mixed	PB	TaqMan	470/476	149	240	81	178	222	76	0.6261
van der Linden 2006	Netherlands	Caucasian	Mixed	PB	RFLP-PCR	99/213	20	51	28	33	99	81	0.7627
Doudney 2009	UK	Caucasian	Mixed	PB	RFLP-PCR	263/184	64	116	83	41	99	44	0.3002
MTHFD1 G1958A													
Hol 1998	Netherlands	Caucasian	Mixed	PB	qRT-PCR	36/335	13	19	4	100	172	63	0.4692
Brody 2002	Ireland	Caucasian	Mixed	PB	qRT-PCR	336/997	101	178	57	283	526	188	0.0406
Scott 2006	Ireland	Caucasian	Mixed	PB	qRT-PCR	176/770	50	84	42	209	411	150	0.0405
De Marco 2006	Italy	Caucasian	SB	HB	RFLP-PCR	142/523	25	74	43	143	251	129	0.3668
Blom 2007	Germany	Caucasian	Mixed	HB	RFLP-PCR	103/203	31	58	14	71	98	34	0.9852
Carroll 2009	Ireland	Caucasian	Mixed	PB	qRT-PCR	509/966	97	250	162	198	468	300	0.5315
FOLH1 T223C													
Das 2018	India	Asian	Mixed	HB	qRT-PCR	62/73	38	23	1	38	25	10	0.0014
DHFR 19 bp del													

Das 2018	India	Asian	Mixed	HB	qRT-PCR	62/73	35	21	6	29	33	11	0.1776
MCM6 C9094T													
Hoang 2019	USA	Caucasian	Mixed	PB	Qiagen multiplex PCR kit	378/421	142	142	94	141	180	140	0
RFC1 A80G													
Marco 2003	Italy	Caucasian	Mixed	PB	RFLP-PCR	161/143	14	30	19	21	35	22	0.3657
Relton 2004	UK	Caucasian	Mixed	PB	RFLP-PCR	206/602	67	93	46	200	304	98	0.3286
Pei 2005	China	Asian	Mixed	PB	RFLP-PCR	104/99	14	46	44	22	50	27	0.8996
Cao 2018	China	Asian	Mixed	HB	MassARRAY	152/169	26	64	62	39	84	46	0.9564
BHMT G716A													
Cao 2018	China	Asian	Mixed	HB	MassARRAY	152/169	58	74	20	81	75	13	0.1366
MS A2756G													
Morrison 1998	UK	Caucasian	Mixed	NR	TaqMan	32/72	19	12	1	48	23	1	0.3365
Christensen 1999	Canada	Caucasian	SB	PB	RFLP	55/97	38	17	0	59	28	10	0.0268
Akar 2000	Turkey	Caucasian	Mixed	PB	NR	56/76	38	17	1	39	32	5	0.6452
Johanning 2000	USA	Caucasian	Mixed	NR	RFLP	77/84	59	18	0	70	13	1	0.6576
De Marco 2002	Italy	Caucasian	Mixed	PB	RFLP	174/210	138	34	2	148	61	1	0.0438
SHMT1 C1420T													
Rebekah 2017	India	Asian	Mixed	HB	PCR-DHPLC	124/184	16	54	54	40	82	62	0.91106
HO-1 A413T													
Fujioka 2015	USA	Caucasian	SB	PB	qRT-PCR	128/131	15	65	48	13	67	51	0.60963
PCMT1													
Wang 2013	China	Asian	Mixed	PB	HRM	116/141	70	37	9	87	45	9	0.44451
