# Supplementary material for: Folate Pathway Gene Single Nucleotide Polymorphisms and Neural Tube Defects: A Systematic Review and Meta-Analysis
Source: J Pers Med. 2022 Sep 29;12(10):1609. doi: 10.3390/jpm12101609 (PMC9605131; doi:10.3390/jpm12101609)
Supplement: Supplementary file 1 [file jpm-12-01609-s001.zip › Supplementary Table S2.pdf]

Table S2.

Papers	Author 1										Author 1									
	Selection				Comparability		Exposure				Selection				Comparability		Exposure			
	Is the case definition adequate?	Representativeness of the Cases	Selection of Controls	Definition of Controls	Comparability of Cases and Controls on the Basis of the Design or Analysis	Demonstration that outcome of interest was not present at start of study	Ascertainment of Exposure	Same Method of Ascertainment for Cases and Controls	Non-Response Rate	Total	Is the case definition adequate?	Representativeness of the Cases	Selection of Controls	Definition of Controls	Comparability of Cases and Controls on the Basis of the Design or Analysis	Demonstration that outcome of interest was not present at start of study	Ascertainment of Exposure	Same Method of Ascertainment for Cases and Controls	Non-Response Rate	Total
van der Put 1998	1	1	1	0	1	1	1	0	0	6	1	1	1	0	1	1	1	0	1	7
De Franchis 1998	1	1	0	0	1	1	1	1	0	6	1	1	1	0	1	1	1	0	1	7
Boduroƒülu 1998	1	1	1	1	1	1	1	0	0	7	1	1	0	0	0	1	0	0	1	4
Garcia-Fragoso 2002	1	1	0	0	1	1	1	0	0	5	1	1	1	0	1	1	1	0	1	7
Perez 2003	1	1	1	0	1	1	1	0	0	6	1	1	1	0	1	1	1	0	1	7
Relton 2003	1	1	0	1	1	1	0	0	0	5	1	1	1	1	1	0	0	0	0	5
Sadewa 2004	0	1	1	1	1	1	1	0	0	6	1	1	1	1	1	1	1	0	1	8
Kirke 2004	1	1	1	0	1	1	1	0	0	6	1	1	1	1	1	0	1	0	0	6
Gao 2009	1	1	1	0	1	1	1	0	0	6	1	1	1	1	1	0	0	0	0	5
Eser 2010	1	1	0	0	1	1	1	0	1	6	1	1	1	1	1	1	1	0	1	8
Harisha 2010	0	1	0	1	1	0	1	0	0	4	1	1	1	1	1	0	1	0	0	6
Wei 2013	0	0	1	1	1	0	0	1	0	4	1	1	1	1	1	1	1	0	1	8

Liu 2013	1	1	1	1	1	1	1	0	0	7	1	1	1	1	1	0	1	0	0	6
Qin 2014	0	0	1	1	1	0	0	0	0	3	1	1	1	1	1	1	1	0	1	8
Yu 2014	1	0	1	1	1	1	0	1	0	6	1	1	1	1	1	0	1	0	0	6
Pardo 2014	0	1	0	1	1	0	1	0	0	4	1	1	1	1	1	1	1	0	1	8
Wang 2015	1	1	1	1	1	1	1	0	0	7	1	1	1	0	1	1	1	0	1	7
Dutta 2017	1	0	0	0	1	1	0	0	0	3	1	1	0	1	0	1	1	0	1	6
Fang 2018	1	0	1	1	1	1	0	0	0	5	1	1	1	1	1	0	1	0	0	6
Stegmann 1999	1	1	1	1	1	1	0	0	0	6	1	1	1	1	1	1	1	0	1	8
Barber 2000	0	0	1	1	1	1	1	1	0	6	1	1	1	0	1	1	1	0	1	7
Volcik 2000	1	1	1	1	1	0	0	0	0	5	1	1	0	1	0	1	1	0	1	6
Richter 2001	0	0	1	1	1	1	0	0	0	4	1	1	1	0	1	0	0	0	0	4
Cunha 2002	1	0	0	0	1	1	1	0	0	4	1	1	1	1	1	1	1	0	1	8
De Marco 2002	0	1	1	1	1	0	0	0	1	5	1	1	1	0	1	1	1	0	1	7
Parle-McDermott 2003	1	1	0	0	1	1	1	0	1	6	1	1	0	1	0	1	1	0	1	6
Felix 2004	1	0	1	0	1	0	0	0	1	4	1	1	1	1	1	0	1	0	0	6
Gos 2004	1	0	0	1	1	1	0	1	1	6	1	1	1	1	1	1	1	0	1	8
Grandone 2006	0	0	1	0	1	0	1	0	1	4	1	1	1	0	1	1	1	0	1	7
Gonzalez-Herrera 2007	0	1	1	1	1	1	1	0	1	7	1	1	0	1	0	1	1	0	1	6

Behunova 2010	0	0	1	1	1	0	1	0	1	5	1	1	1	0	1	0	0	0	0	4
Selvi 2010	1	1	1	1	1	0	1	0	1	7	1	1	0	1	0	1	1	0	1	6
Yildiz 2016	0	0	1	1	1	1	0	0	0	4	1	1	1	1	1	0	1	0	0	6
van der Put 1997	1	0	1	1	1	1	1	0	1	7	1	1	1	1	1	1	1	0	1	8
Morrison 1998	0	0	1	1	1	0	1	0	1	5	1	1	1	0	1	1	1	0	1	7
Christensen 1999	1	1	1	1	1	0	1	1	1	8	1	1	0	1	0	1	1	0	1	6
Shaw 1999	0	0	1	1	1	0	0	0	1	4	1	1	1	1	1	0	1	0	0	6
Johanning 2000	1	0	1	1	1	0	0	0	1	5	1	1	1	1	1	1	1	0	1	8
O'Leary 2005	0	1	0	1	1	0	1	0	1	5	1	1	1	0	1	1	1	0	1	7
Doudney 2009	1	1	1	1	1	0	1	0	1	7	1	1	0	1	0	1	1	0	1	6
Wilson 1999	1	0	1	1	1	1	1	0	1	7	1	1	1	0	1	0	0	0	0	4
Pietrzyk 2003	1	0	1	1	1	1	0	1	0	6	1	1	0	1	0	1	1	0	1	6
Relton 2004	0	0	0	1	1	1	1	0	1	5	1	1	1	1	1	0	1	0	0	6
van der Linden 2006	1	1	1	0	1	1	1	0	0	6	1	1	1	1	1	1	1	0	1	8
Hol 1998	1	1	0	0	1	1	1	1	0	6	1	1	1	0	1	1	1	0	1	7
Brody 2002	1	1	1	1	1	1	1	0	0	7	1	1	0	1	0	1	1	0	1	6
Scott 2006	1	1	0	0	1	1	1	0	0	5	1	1	1	1	1	0	1	0	0	6
De Marco 2006	1	1	1	0	1	1	1	0	0	6	1	1	1	1	1	1	1	0	1	8

Blom 2007	1	1	0	1	1	1	0	0	0	5	1	1	1	1	1	0	1	0	0	6
Carroll 2009	0	1	1	1	1	1	1	0	0	6	1	1	1	1	1	1	1	0	1	8
Das 2018	1	1	1	0	1	1	1	0	0	6	1	1	1	1	1	0	1	0	0	6
Hoang 2019	1	1	1	0	1	1	1	0	0	6	1	1	1	0	1	0	1	0	1	6
Marco 2003	1	1	0	0	1	1	1	0	1	6	1	1	1	0	1	1	1	0	1	7
Pei 2005	0	1	0	1	1	0	1	0	0	4	1	1	0	1	0	1	1	0	1	6
Cao 2018	0	0	1	1	1	0	0	1	0	4	1	1	1	1	1	0	1	0	0	6
Akar 2000	1	1	1	1	1	1	1	0	0	7	1	1	1	1	1	1	1	0	1	8
Rebekah 2017	0	0	1	1	1	0	0	0	0	3	1	1	1	0	1	1	1	0	1	7
Fujioka 2015	1	0	1	1	1	1	0	1	0	6	1	1	1	1	1	1	1	0	1	8
Wang 2013	0	1	0	1	1	0	1	0	0	4	1	1	1	0	1	0	0	0	0	4
