# Supplementary figures and images for: Folate Pathway Gene Single Nucleotide Polymorphisms and Neural Tube Defects: A Systematic Review and Meta-Analysis
Source: J Pers Med. 2022 Sep 29;12(10):1609. doi: 10.3390/jpm12101609 (PMC9605131; doi:10.3390/jpm12101609)

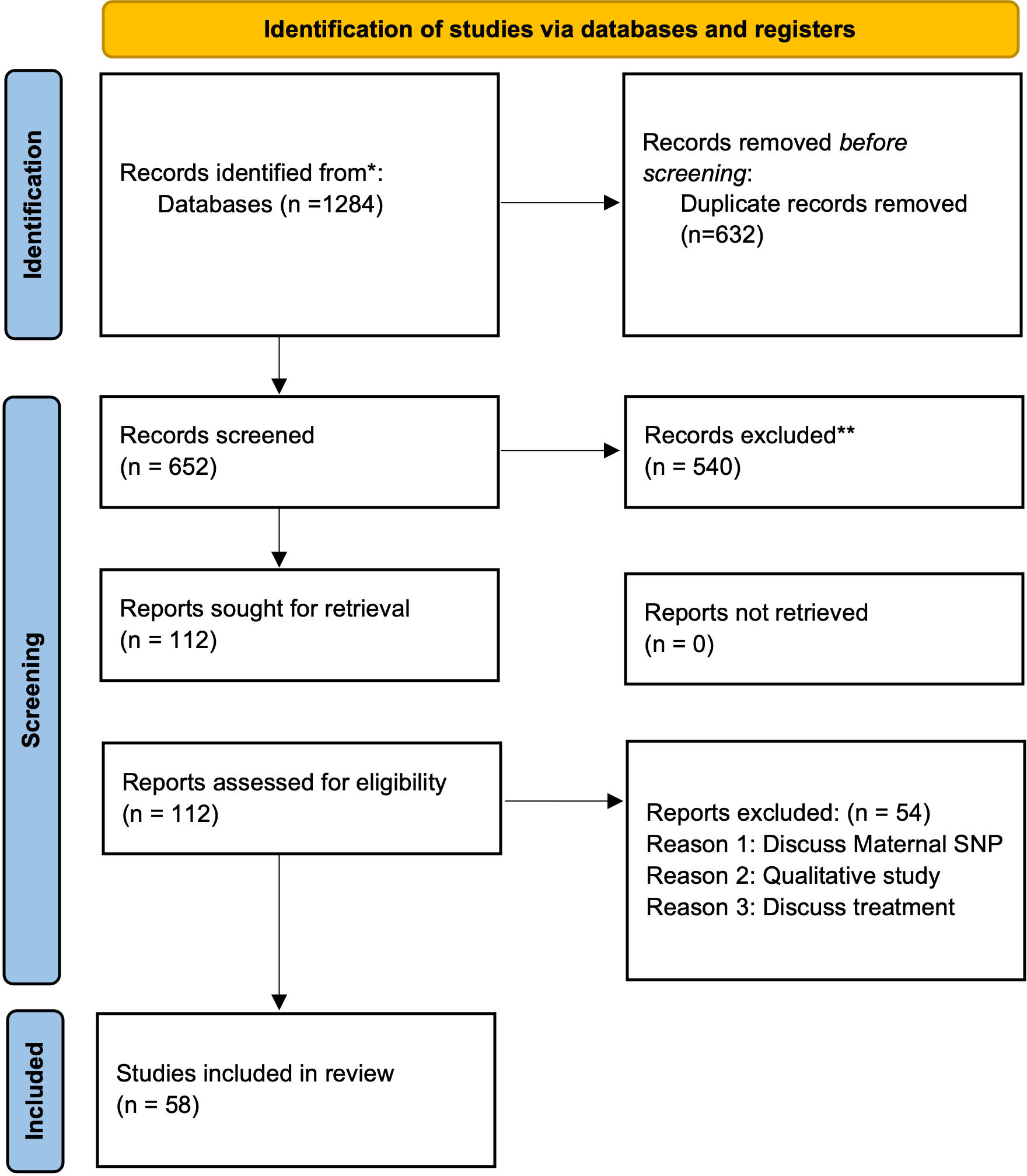

Supplement: Supplementary file 1 [file jpm-12-01609-s001.zip › Supplementary Figure S1.png]

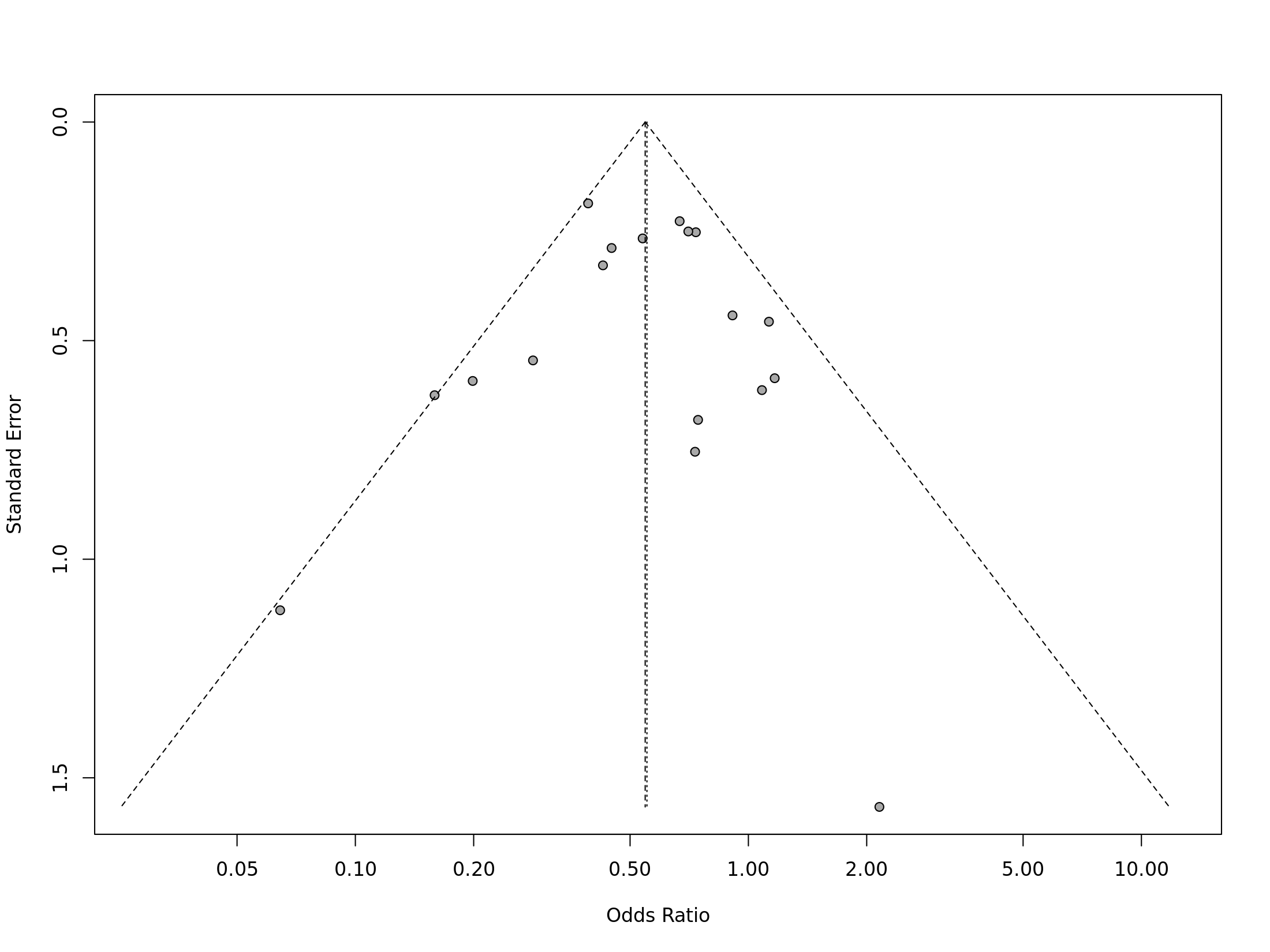

Supplement: Supplementary file 1 [file jpm-12-01609-s001.zip › Supplementary Figure S2.png]

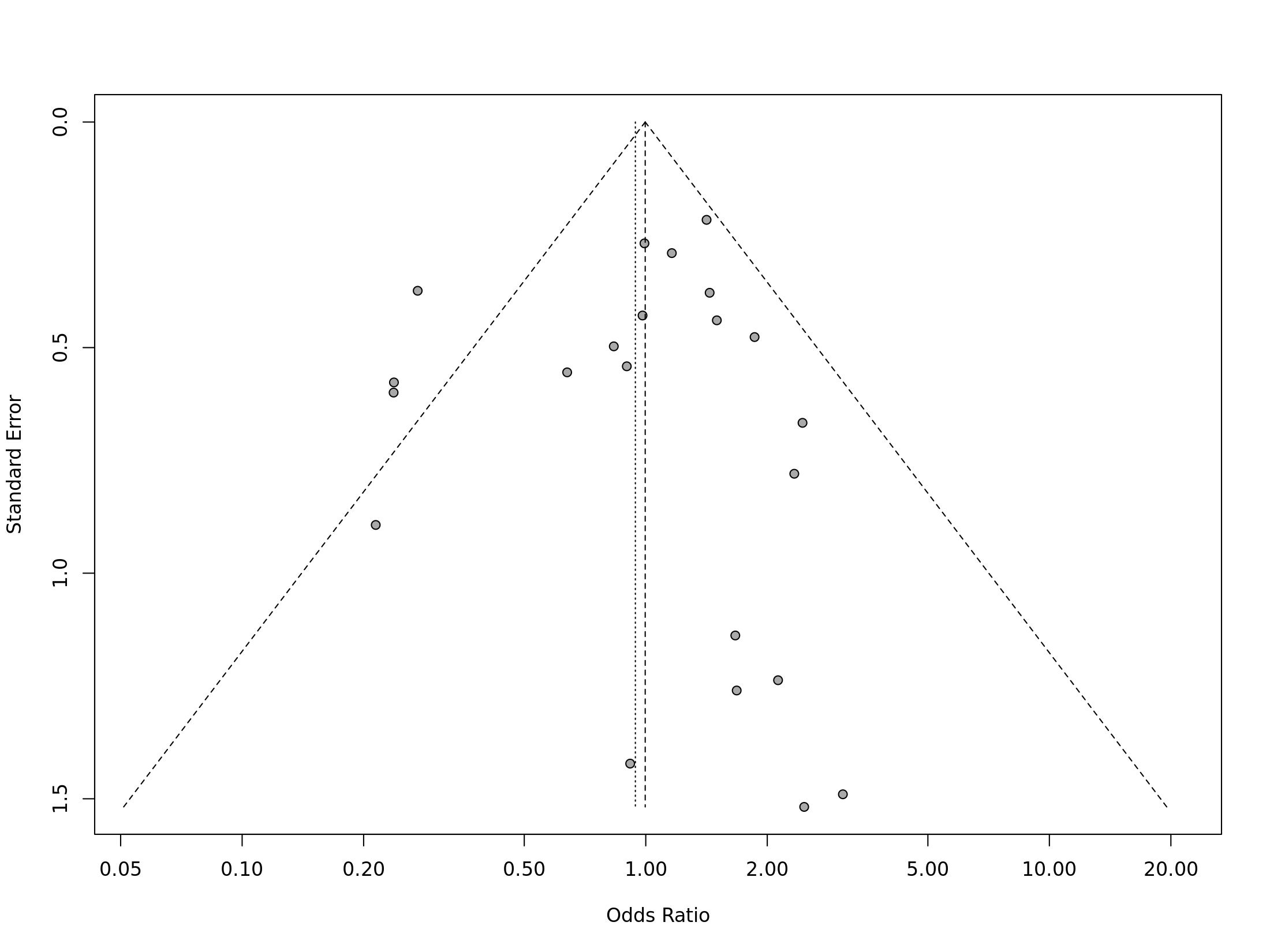

Supplement: Supplementary file 1 [file jpm-12-01609-s001.zip › Supplementary Figure S3.png]

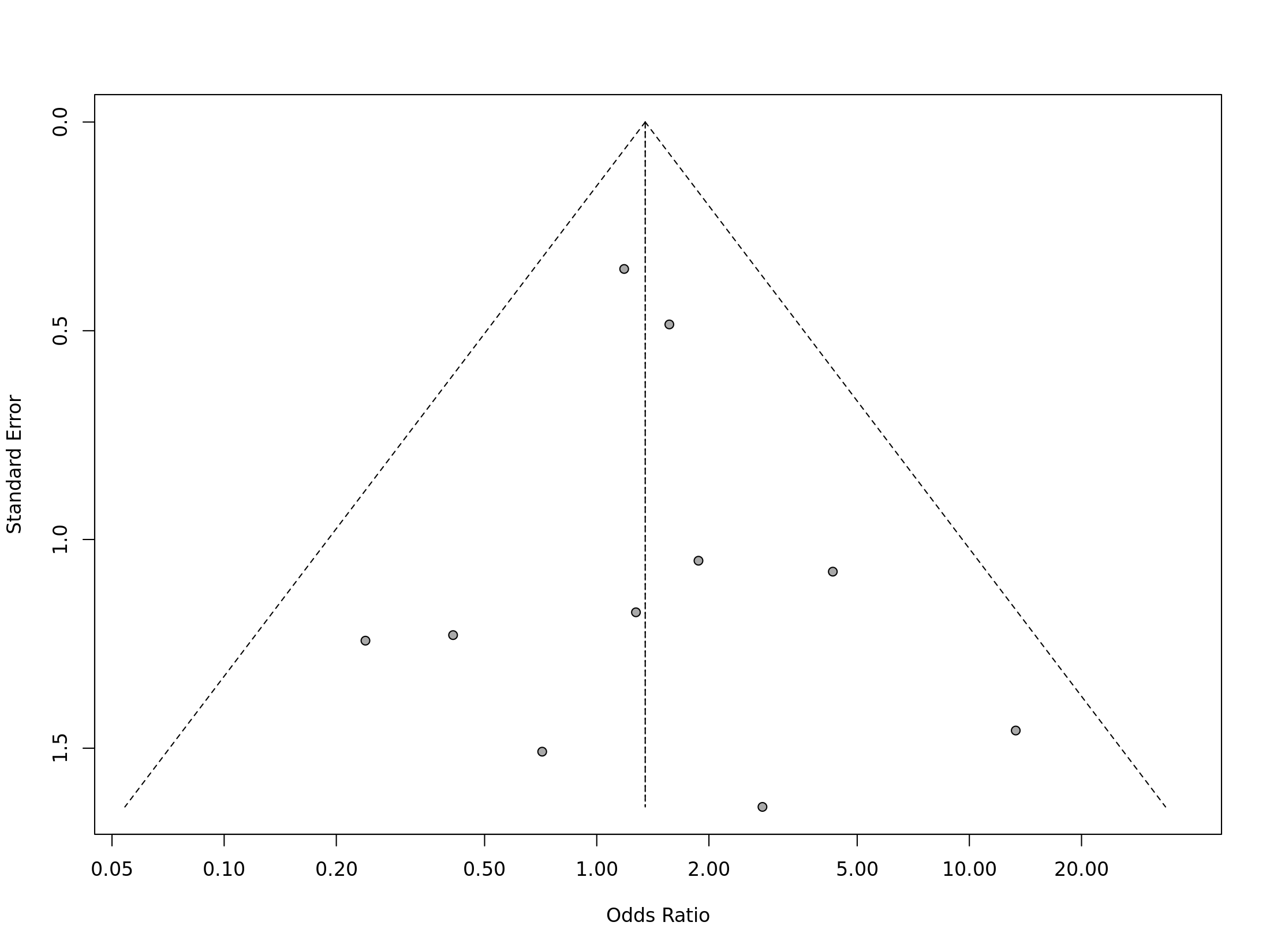

Supplement: Supplementary file 1 [file jpm-12-01609-s001.zip › Supplementary Figure S4.png]

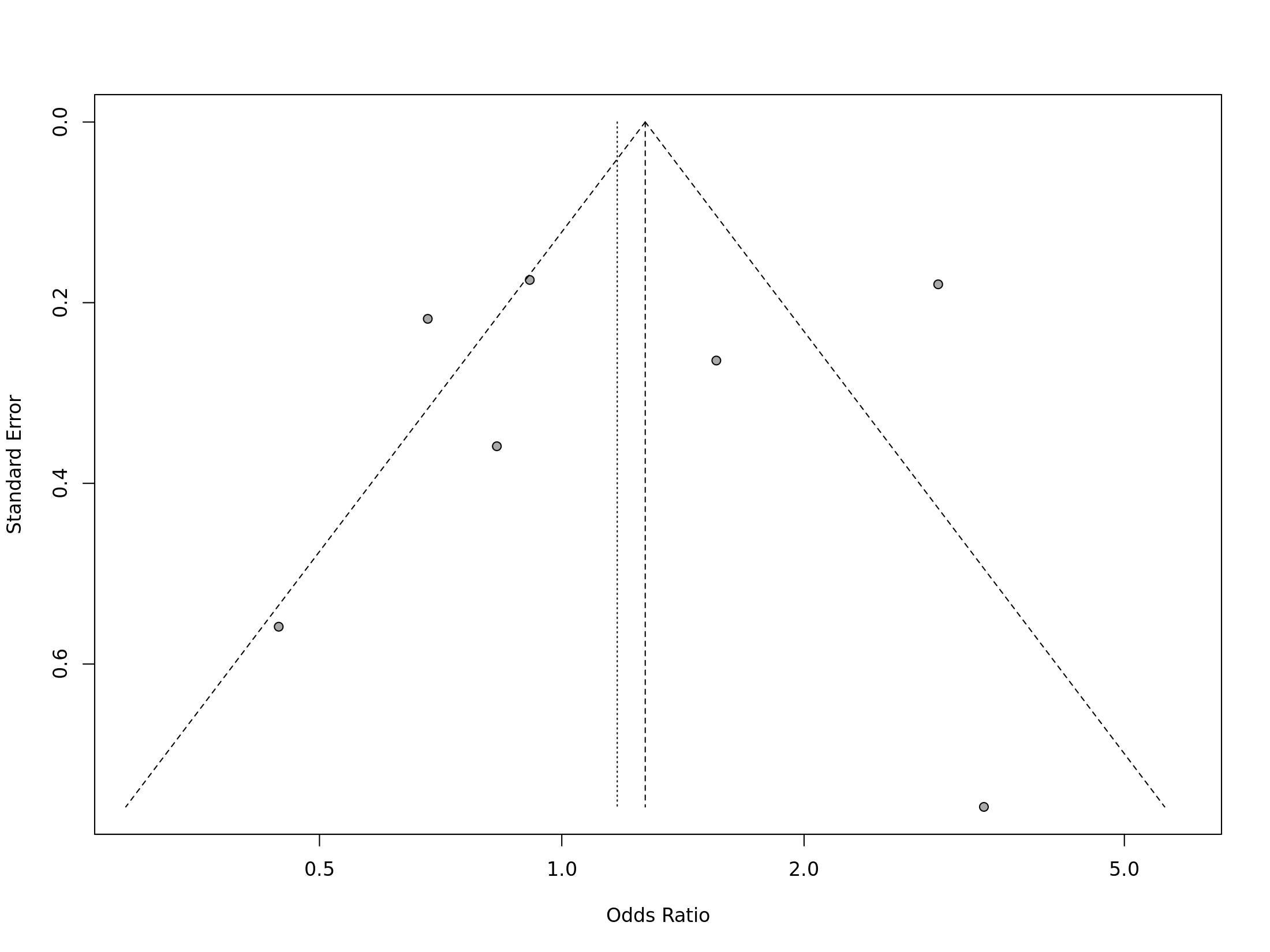

Supplement: Supplementary file 1 [file jpm-12-01609-s001.zip › Supplementary Figure S5.png]

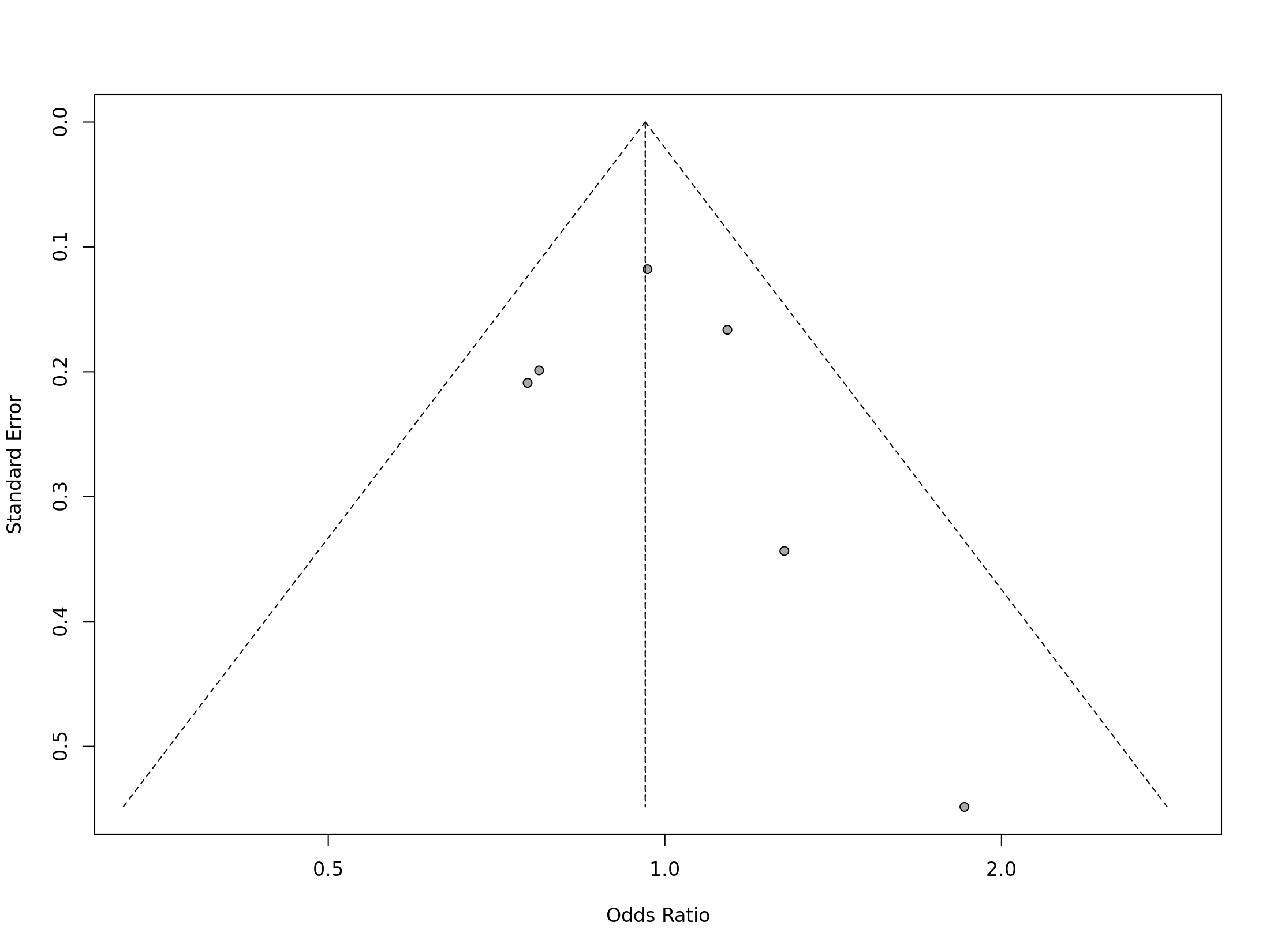

Supplement: Supplementary file 1 [file jpm-12-01609-s001.zip › Supplementary Figure S6.png]

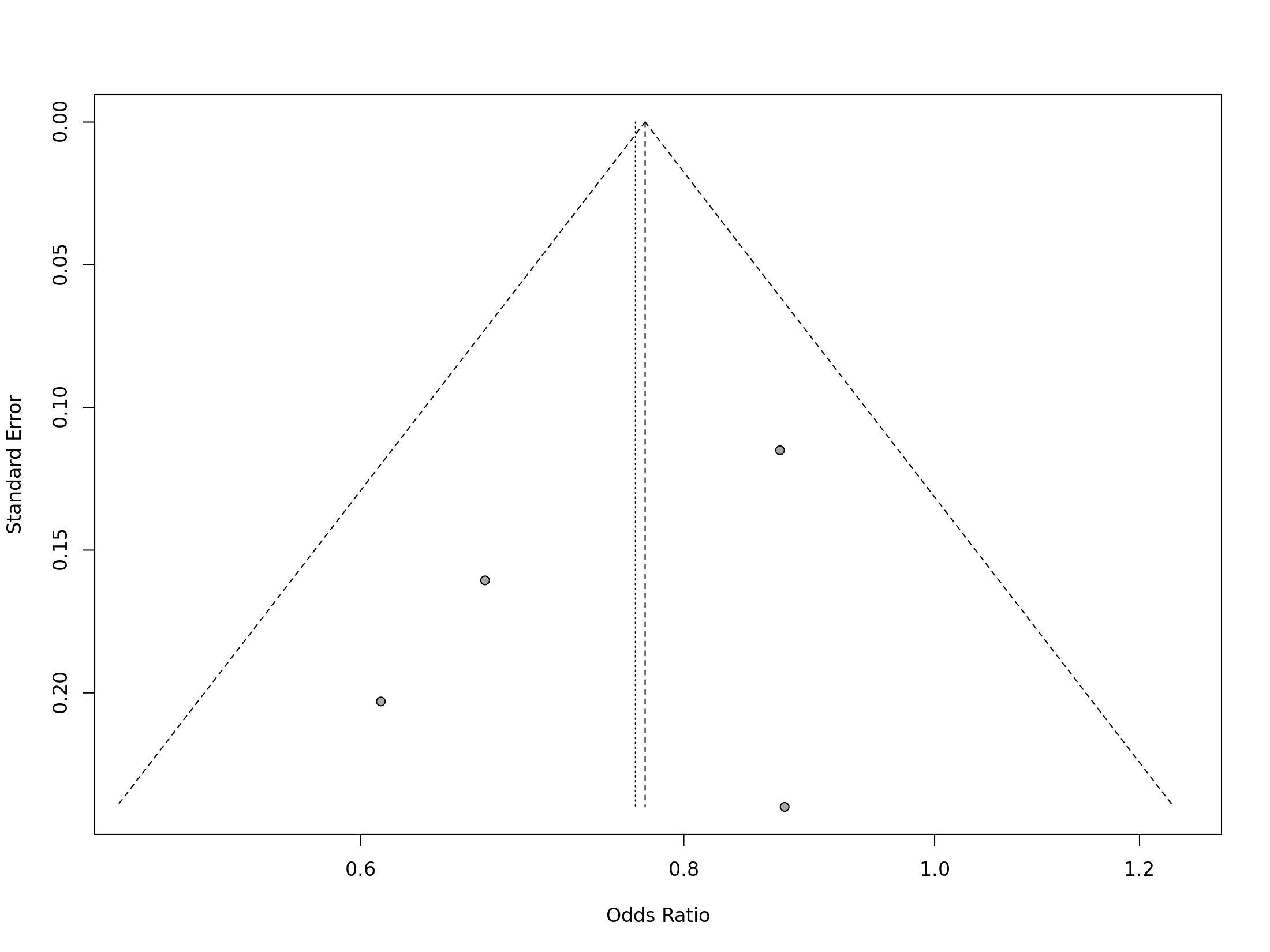

Supplement: Supplementary file 1 [file jpm-12-01609-s001.zip › Supplementary Figure S7.png]
